# 3D Bioprinting for Next-Generation Personalized Medicine

**DOI:** 10.3390/ijms24076357

**Published:** 2023-03-28

**Authors:** Ethan Hau Yin Lam, Fengqing Yu, Sabrina Zhu, Zongjie Wang

**Affiliations:** 1 Faculty of Arts and Science, University of Toronto, Toronto, ON M5S 3G3, Canada; 2 Department of Pharmacology & Toxicology, University of Toronto, Toronto, ON M5S 1A8, Canada; 3 Department of Nutritional Sciences, University of Toronto, Toronto, ON M5S 1A8, Canada; 4 Department of Computer Science, University of Toronto, Toronto, ON M5S 1A4, Canada; 5 Department of Laboratory Medicine and Pathobiology, University of Toronto, Toronto, ON M5S 1A8, Canada; 6 Leslie Dan Faculty of Pharmacy, University of Toronto, Toronto, ON M5S 3M2, Canada; 7 Institute of Biomedical Engineering, University of Toronto, Toronto, ON M5S 3E1, Canada; 8 McCormick School of Engineering, Northwestern University, Chicago, IL 60611, USA

**Keywords:** bioprinting, biomaterial, drug discovery, personalized medicine, precision medicine, regenerative medicine, stem cell

## Abstract

In the past decade, immense progress has been made in advancing personalized medicine to effectively address patient-specific disease complexities in order to develop individualized treatment strategies. In particular, the emergence of 3D bioprinting for in vitro models of tissue and organ engineering presents novel opportunities to improve personalized medicine. However, the existing bioprinted constructs are not yet able to fulfill the ultimate goal: an anatomically realistic organ with mature biological functions. Current bioprinting approaches have technical challenges in terms of precise cell deposition, effective differentiation, proper vascularization, and innervation. This review introduces the principles and realizations of bioprinting with a strong focus on the predominant techniques, including extrusion printing and digital light processing (DLP). We further discussed the applications of bioprinted constructs, including the engraftment of stem cells as personalized implants for regenerative medicine and in vitro high-throughput drug development models for drug discovery. While no one-size-fits-all approach to bioprinting has emerged, the rapid progress and promising results of preliminary studies have demonstrated that bioprinting could serve as an empowering technology to resolve critical challenges in personalized medicine.

## 1. Introduction

Therapeutic strategies have traditionally been developed for a broad population of patients that have been primarily categorized using pathological observations and clinical features [1]. However, various therapeutic strategies, including medications, are currently being designed to recognize variable efficacy and adverse effects in patients depending on differing lifestyles, genomic signatures, and additional comorbidities [1,2]. The genetic variability of patients also leads to the risk of organ rejection in regenerative medicine and organ transplantation (notably, HLA and minor histocompatibility antigen mismatch between donor and recipient), which has slowed the translation from an in vitro disease model to further testing in the clinical patient-care setting [3]. Given that disease states are impacted by a complex combination of factors, there is a significant need to individualize patient treatments. Personalized medicine is a rapidly advancing field of health care that uses an individual’s unique genetic profile to direct prevention, diagnosis, and treatment [2]. It aims to tailor and individualize therapeutic treatment plans according to the patients’ physiology, genetic profile, and drug response [2]. As a result, patients may receive a treatment with optimal efficacy and dosage, which overall enhances patient safety and clinical outcomes by avoiding misdiagnosis and adverse drug effects. Industries are also likely to benefit from reduced healthcare costs and favorable patient outcomes [4].

**Figure 1:**
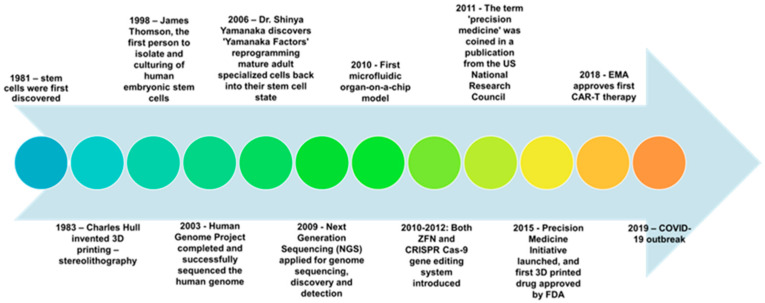
A brief timeline of major scientific discoveries and events [5,6,7,8,9,10,11,12,13,14,15,16,17].

Personalized medicine has undergone multiple breakthroughs in the last several decades. These major discoveries are summarized in Figure 1. For example, the discovery of stem cells, especially human-induced pluripotent stem cells (hiPSC) by the Yamanaka factors [5] revolutionized the field of disease treatments, as they provided a method to generate induced pluripotent stem cells (iPSCs) without the use of embryos, which eliminated ethical concerns [6]. Currently, there are numerous iPSC-derived cell products and grafts in the pipeline of clinical translation. In addition to biological discovery, engineering technologies have also played an important role in the breakthrough of personalized medicine. The world’s first 3D-printeYes, d drug, Spritam, was approved by the U.S. Food and Drug Administration (FDA) for the treatment of partial-onset seizures for epilepsy [7].

While major advancements and technologies in precision medicine have been accomplished within the last few decades that have identified more sensitive and specific biomarkers and have better mediated drug dosages and selection research, challenges restricting the potential of personalized medicine remain [2]. The difficulties mainly lie in the validation of experimental results, accessibility of patient data, and transferability to the clinical setting. While numerous of genetic markers have been discovered, it was reported in the 2008 PCAST that the clinical validation of these candidate markers has been proceeding at a slower pace [8]. Many selected therapeutic agents that were successful in models could not be readily adapted for the clinical context and have failed because of drug intolerance, highlighting the need for accurate and representative pre-clinical models [9]. As patient samples are often related to ethical, legal, and social issues regarding accessibility, there has been a focus on developing patient-derived models, such as immortalized cell lines, patient-derived xenografts (PDXs), and organoids. As compared to traditional monolayer cultures, PDXs and organoids are better at mimicking in vivo micro-environments and, thus, have improved the generalizability of experimental outcomes to clinical contexts [9,10]. However, they have been limited due to their time-intensive requirements and significant costs [10]. Moreover, there has been a lack of consistency in the components required and processes involved in organoid protocols among different researchers, which has resulted in unexpected variabilities when comparing their results [9]. Therefore, developing high-throughput, consistent, and representative experimental models is important to facilitate personalized therapy and maximize treatment efficacy.

Recently, 3D bioprinting has emerged as a potential method for resolving the aforementioned challenges. The fabrication of 3D biological structures with multiple functional, structural, and mechanical components and properties is the core of 3D bioprinting, and this is performed through the precise, layer-by-layer positioning of biological materials, biochemicals, and living cells [11]. As a result, 3D cultures created by bioprinting technologies have been used to facilitate drug development and toxicity, as well as the study of tissue and disease formation and progression [12]. In particular, its application for in vitro tissue and organ modeling that is capable of mimicking human physiology presents novel opportunities, such as personalized implants and drug development, to improve personalized medicine. While 3D bioprinting is an exciting potential tool for advancing personalized medicine, the existing bioprinted constructs are not yet able to fulfill the ultimate goal: an anatomically realistic organ with mature biological functions. Current bioprinting approaches still have technical challenges in terms of precise cell deposition, effective differentiation, proper vascularization, and innervation.

This review provides an overview of the current developments in bioprinting techniques and applications and assesses the potential of 3D bioprinting to advance personalized medicine. Established bioprinting techniques, with an emphasis on extrusion printing and digital light processing (DLP), and available bioprinting materials, such as induced pluripotent stem cells, were considered. Major applications for bioprinted constructs, including the engraftment of stem cells as personalized implants for regenerative medicine, and in vitro high-throughput drug development models for drug discovery were evaluated. Finally, we summarized the current state-of-the-art in bioprinting, as well as the key challenges, and concluded with a brief perspective of bioprinting in personalized medicine.

## 2. Bioprinting: Methods and Materials

**Figure 2:**
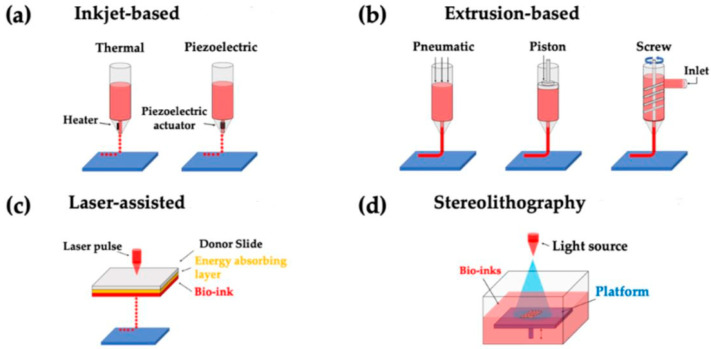
Four main bioprinting methods. (**a**) Inkjet Bioprinting. (**b**) Extrusion Bioprinting. (**c**) Laser-Assisted Bioprinting. (**d**) Stereolithography Bioprinting were reprinted with permission from Ref. [14]. 2020, Yu. This figure is modified, and obtained from an open-access journal article.

Bioprinting consists of two important aspects: the printing methods and the corresponding materials. This section reviews the concepts and advancements within these aspects to achieve optimized performance. There are four main bioprinting technologies: inkjet, laser-assisted, extrusion, and stereolithography (Figure 2). While each bioprinting technology has both strengths and limitations, each may be utilized according to the intended application to further develop the field of personalized medicine. A detailed comparison of different bioprinting methods is provided in Table 1 and in reference [13].

### 2.1. Bioprinting Technology

#### 2.1.1. Inkjet Printing

Inkjet printing is the first bioprinting technology with a cartridge that stores bio-ink. This method is generally known for its affordability and easy accessibility, as commercial printers can be easily modified to accommodate the technology [13,14,15,16]. It has also demonstrated high cell viability; approximately 80–90% of cells remain functional [16]. This process utilizes a thermal or piezoelectric actuator to generate droplets of the bio-ink consistently.

The thermal actuator utilizes heat-induced bubble nucleation that causes pressure to build-up for the expulsion of the droplet. Thermal-based inkjet bioprinters typically reach temperatures of 100–300 °C. As a result of this high heat, this method of inkjet printing can cause cellular stress in the bio-ink [17]. However, it may be possible to mitigate the cellular stress factor by localizing the high temperatures on the cell for a minimal amount of time [18,19]. Alternatively, piezoelectric inkjet bioprinters, which produce acoustic waves at low viscosities and concentrations, have been used [20].

#### 2.1.2. Extrusion Printing

Extrusion-based bioprinting is a modification of inkjet printing that exerts a constant force on the bio-ink during output [21]. This results in a cylindrical printed stream that attaches to the intended surface as a continuous line [22,23]. This is a significantly different approach to the singular, high-cell-density, bio-ink droplets found in standard inkjet bioprinting [24]. The limitations of this technique include a reduced cell viability due to the exposure of bio-inks to further mechanical stress and having a limited resolution over 100 µm [25].

#### 2.1.3. Laser-Assisted Printing

Laser-assisted printing is a form of bioprinting that propels bio-ink onto the printing surface using a high-pressure bubble [20,21]. The formation of this bubble begins with a “donor layer”, also known as a ribbon. It contains an energy-absorbing layer, which is subjected to a high-energy laser pulse that vaporizes the layer at the focal spot [24]. The donor layer, which is composed of a thin layer of glass, metal, and bio-ink, is ultimately vaporized into a high-pressure bubble that enables the bio-ink to be propelled onto the printing surface [26]. Koch and their colleagues demonstrated that the jet velocity has been shown to be higher for smaller focal spots and dependent on the laser intensity. More specifically, at a laser intensity of 1.4 J/cm^2^, the jet velocity on a spot size of 4000 μm^2^ was higher, as compared to the velocity at 3000 μm^2^ [27]. This bioprinting method has numerous advantages. It promotes excellent cell viability at a high resolution, between 10–50 µm [11], while also avoiding contact between the dispenser nozzle and the bio-ink. However, this printing method is expensive, and the long-term effects of the laser have yet to be fully explored [24].

#### 2.1.4. Stereolithography

Stereolithography utilizes visible or ultraviolet (UV) light via DLP to solidify bio-ink in a layer-by-layer process [24,25,26,27]. This process is known for eliminating shear pressure during nozzle-based printing due to its rapid, highly precise resolution, with a resolution range of 5–300 µm [26]. The exposure of light ultimately results in the solidification of the layers via photopolymerization [28]. For example, Gauvin et al. fabricated 3D structures utilizing a gelatin-based prepolymer solution under UV light [29]. However, the UV light exposure placed the bio-ink cells at risk of cytotoxic damage from photo-initiators, which could negatively impact cell viability [26,30]. Researchers have attempted to solve this problem by adding two monomers of alkene or thiol groups that could react spontaneously under UV irradiation at 266 nm. After 3 days, researchers observed that the cell viability was much higher, than the control variable, at approximately 95% [31].

Advancements in personalized medicine have been limited primarily by the expense of using 3D bioprinters. Furthermore, better technology and more precise bioprinting is likely to result in even higher costs. This ultimately makes more costly options, such as laser-assisted printers, inaccessible to medical laboratories and institutes, which in turn limits the advanced research on laser-assisted printing in personalized medicine.

**Table 1:** Comparison of four major types of bioprinting techniques.

Bioprinting Method	Key Aspects	Advantages	Disadvantages	References
Inkjet	First bioprinting technology that has a bio-ink cartridge. Minimum droplet volume of 20 nL	Low cost Easy accessibility High cell viability (>85%)	Thermal actuator is potentially prone to high-temperature stress	[13,14,15,16]
Extrusion	A modification of inkjet-based bioprinting that prints a cylindrical stream onto a printing surface in a continuous line	Highly controlled printing structure	Limited resolution (100–500 µm) Reduced cell viability	[22,23,32]
Laser-assisted	Propels bio-ink onto the printing surface	High cell viability (>95%) Fast printing speed High resolution (10–50 µm)	High cost Long-term effects of laser unclear	[11,20,21,33]
Stereolithography	Uses UV light to solidify bio-ink layer-by-layer	Fast printing speed High resolution Excellent cell adhesion	High cost UV light exposure could reduce cell viability via cytotoxic damage	[24,25,27,34]

Note: More information is provided in Table 1 of reference [13].

### 2.2. Cell Source and Bio-Inks

In addition to printing technologies, the formulation of bio-inks, usually in the combination of supportive biomaterials and specific cell types, is critical for the success of bioprinting. The biomaterials used in bioprinting can be roughly divided into two subtypes: (1) natural biomaterials, such as alginate [14,35,36,37], agarose [35,38], collagen [39,40], and nanocellulose [41]; and (2) synthetic biomaterials, such as polyethylene glycol diacrylate (PEDGA) [42], and Pluoronic^®^ [43,44]. Each material has unique mechanical (e.g., printability) and biological properties (e.g., the ability to support long-term cell adhesion and growth). A detailed comparison of the different bioprinting methods is provided in Table 2 and in reference [13].

The cell source for bioprinting is an important factor. To fabricate complex tissues and organs that mimic their natural counterparts, the cells for bioprinting need to not only reproduce the desired biological function but also to expand as required, as overexpansion could result in hyperplasia or cell death [45]. The difficulty of culturing many primary cells and their limited lifespans has made reproducing them via bioprinting infeasible [45], and the intrinsic abnormalities and mutations found in immortalized cell lines of tissues has limited their application as well [46]. Therefore, there has been increased interest using stem cells in 3D bioprinting.

Stem cells have excellent therapeutic potential because of their unique self-renewal and pluripotency. Due to the ethical concerns regarding embryonic stem cells, researchers have identified methods to induce pluripotency by reprogramming human somatic cells [5,47]. Bioprinting with these hiPSCs has unique advantages for regenerative and personalized medicine applications, such as patient-specific disease modeling, personalized implants, and drug development. This section introduces the current progress in bioprinting using stem cells and the application for personalized implants.

**Figure 3:**
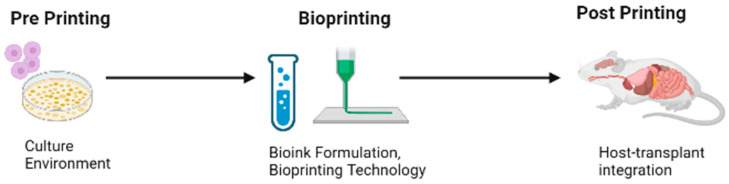
A schematic of factors under consideration for the bioprinting of stem cells.

Human pluripotent stem cells are sensitive to environmental parameters [48], and thus, maintaining cell viability, pluripotency, and differentiation during and after the printing process is of high interest (Figure 3). Many researchers have studied different types of bioprinting and their effects on bio-inks to identify optimal printing methods. For example, both extrusion printing and laser printing did not affect the survival and pluripotency of hiPSCs [48,49]. There have also been novel technologies, such as a “microscopic painting device using a painting needle method” that improved the printing resolution while maintaining the high viscosity of the bio-ink and cell viability [50].

The cell-laden matrix, or the bio-ink, is another factor that influences stem cells [51,52,53]. Several studies confirmed that the use of Matrigel as a hydrogel could maintain pluripotency [48,54]. There have also been advancements in bio-ink formulations. Crook et al. used a clinically amenable bio-ink that was cross-linked to a 3D construct in order to maintain the multilineage cell-induction potential of iPSCs [55]. Cofiño et al. created a new formulation of bio-ink involving a methylcellulose and RAD16-I-based biomaterial that had high printability and biocompatibility with embedded human mesenchymal stem cells, resulting in high viability and differentiation [56]. Researchers proposed a core-sheath multilayer cell-laden structure that combined a cell-laden collagen-based bio-ink for the core and pure alginate for the sheath, and it protected cells and achieved efficient differentiation [57]. Additionally, there has been increasing interest in decellularized extracellular matrix (dECM) bio-inks [58,59], which create a more natural micro-environment and are likely enhance cellular function. Jang et al. showed that heart-tissue-derived dECM bio-ink provided benefits for the maturation of cardiac progenitor cells (CPC) and improved the epicardial activation, as compared with collagen bio-ink [60].

## 3. Applications in the Discovery of Personalized Medicine

With the aid of advanced bio-inks and 3D bioprinting technology that can maintain the viability and potency of cellular materials, such as stem cells, there have been successful applications in tissue regeneration and drug-development models. This section presents examples of bioprinting used to regenerate tissue for bones, livers, hearts, corneas, and other tissues required by the central neural system, highlighting factors such as cell viability, functionality, proliferation, maturation, and in vivo transplantation. In addition, the application of bioprinting for the development of biomimetic platforms for drug development is introduced. Examples of 3D-bioprinted cardiac tissue models, hepatic models, and glioblastoma models are presented to illustrate the potential of 3D bioprinting in advancing drug development in personalized medicine. The selected major discoveries are summarized in Table 3.

### 3.1. Printing of Stem-Cell Differentiated Organs for Tissue Regeneration

#### 3.1.1. Bone

Currently, 3D bioprinting is a promising approach for bone and cartilage regeneration [61]. In bone tissue engineering, a scaffold has been constructed as a bone substitute that provided mechanical support and facilitated cellular activities, such as migration, proliferation, and differentiation. Poly(lactide) (PLA) has become one of the most used biomaterials for scaffolds, as it is biocompatible and biodegradable. Researchers combined 3D PLA scaffolds with human gingival mesenchymal stem cells (hGMSCs) and extracellular vesicles (EVs) to test their cytotoxicity and regeneration effects [62]. The PLA degradation of byproducts did not induce a cytotoxic response. After six weeks of in vivo implantation in rats subjected to cortical calvaria bone tissue damage, new bone nodules and blood vessels were observed in the calvariae [62]. Additionally, Teixeira et al. further showed an improvement in the osteo-inductivity of 3D-printed PLA scaffolds by incorporating polydopamine (PDA) and type-I collagen as surface coatings [63].

#### 3.1.2. Kidney

The bioengineering of kidneys has been challenging due to the organs’ complex development, spatial organization, and lineage specifications [64]. Studies have shown that in vitro generated 3D cellular aggregates have superior long-term stability, as compared to 2D differentiation [65]. This agreed with findings by Goulart et al. [66], where bioprinting hepatic tissues using human liver iPS-derived parenchymal cells as 3D spheroids enhanced their cellular survival and function, as compared with in vitro single-cell dispersions [67].

**Table 3:** Summary of selected major discoveries in bioprinting applications.

Target Tissue	Bioprinting Method	Cell Type	Biomaterial	Cellular Response	References
Bone	Commercial fused-filament fabrication 3D printer (DeltaWASP 2040; CSP srl, Massa Lombarda, Italy)	Human gingival mesenchymal stem cells (hGMSCs)	Poly(lactide) (PLA), extracellular vesicles (EVs), polyethyleneimine (PEI)-engineered EVs (PEI-EVs)	(1) Both 3D-PLA + EVs + hGMSCs and 3D-PLA + PEI-EVs + hGMSCs showed no cytotoxicity (2) Better osteogenic properties were observed in 3D-PLA + PEI-EVs + hGMSCs. New bone nodules and blood vessels were observed in calvariae after in vivo implantation in rats subjected to cortical calvaria bone tissue damage.	[62]
	3D Cloning FDM printer (Microbras, Brazil), PLA white commercial filament (1.75 mm in diameter, produced by E-Sun, China)	Porcine bone marrow stem cells (MSCs)	Poly(lactic acid) (PLA), polydopamine (PDA), type-I colla-gen (COL I)	PDA combined with COL coating increased cell adhesion and the metabolic activity of MSCs in the early stage (<7 days) of cell culture and facilitated the deposition of extracellular matrix by day 14, and produced much higher amounts of alkaline phosphatase than un-coated PLA by day 21.	
Kidney and Liver	Extrusion bioprinting (Cellink INKREDIBLE + 3D bioprinter)	iPS-derived parenchymal (hepatocyte-like) cells, iPS-derived hepato-cyte-like cells spheroids	Matrigel	Liver constructs from 3D printing with hepatic spheroids showed prolonged survival, reduced cell death, increased urea production, and prolonged secretion of albumin and A1AT, as compared to printed constructs using single-cell dispersion.	[66]
	Extrusion bioprinting (Novogen 3D bio-printer)	iPSC	STEMdiff APEL and TESR-E6 medium	Bioprinted line conformation increased nephron numbers, as measured by an increase in MAFB+ glomerular area, as compared to manual organoids.	[68]
Heart	Spheroid bioprinting with microfluidic-chip-based 3D cell-culturing system (Regenova, Cyfuse Bio-medical K.K., Tokyo, Japan)	Human-induced pluripotent stem-cell-derived cardiomyocytes (hiPSC-CMs), human adult ventricular cardiac fibroblasts (FBs), and human umbilical vein endothelial cells (ECs)	Free of biomaterials	In vivo implantation of the 3D-bioprinted cardiac patches onto nude rat hearts showed viable cells in the patch along with erythrocytes (evidence of vascularization), and the presence of human nucleic acid (HNA)-positive cells in rat myocardium (evidence of engraftment).	[69]
	Spheroid bioprinting with microfluidic-chip-based 3D cell-culturing system (Regenova, Cyfuse Biomedical K.K., Tokyo, Japan)	Human IPSC-derived cardiomyocytes, fibroblasts, and endothelial cells	Free of biomaterials	In vivo implantation of the bioprinted cardiac patches onto rat myocardial infarction model showed lower scar area, higher vessel count, and higher cardiac output than the control group without the implantation. The survival rates were 100% and 83% in the experimental and the control groups, respectively, after 4 weeks of surgery	[70]
Nerve	Micro-extrusion bioprinting	Frontal cortical human neural stem cells (hNSCs)	Polysaccharides alginate (Al), carboxymethyl-chitosan (CMC), and agarose (Ag)	Co-printing of cells with bio-ink allowed the formation of a porous 3D-scaffold encapsulation of stem cells for in situ expansion and differentiation. Differentiated neurons formed synaptic contacts and showed spontaneous calcium spikes and bicuculline-induced bursting activity.	[71]
	Extrusion bioprinting	Cortical neurons and glial cells de-rived from human iPSCs	Matrigel and alginate	Long-term survival of neurons, up to 70 days post-printing, was observed. Functional analysis showed calcium activity and a small degree of synchronous activity.	[49]
	Lab-on-a-printer (LOP) technology (Aspect Biosystems’ RX1 printer)	hiPSC-derived neural progenitor cells	Fibrinogen base with alginate, cross-linked with a mixture of chitosan, calcium chloride, thrombin, and genipin	Cell viability was 91.65 ± 6.85% by day 6 of the culture period, and 64.12 ± 21.27% by day 15. The printed neural tissues showed neurite extension and the expression of neuronal marker TUJ1 and nucleated cell marker	[72,73]
	Extrusion bioprinting	Induced pluripotent stem cell (iPSC)-derived spinal neuronal progenitor cells (sNPCs) and oligodendrocyte progenitor cells (OPCs)	Matrigel	Cell viability was >75% for both iPSC-derived sNPCs and OPCs printed in 50% Matrigel after 4 days in culture. The bioprinted sNPCs differentiated and showed progressive axon propagation in the micro-scale scaffold channels. Functionality was verified by cellular response signaling molecules, potassium and glutamate.	[54]
Pancreas	Micro-extrusion bioprinting	Human umbilical vein endothelial cells	Pancreatic tissue-derived dECM (pdECM)	PdECM increased the insulin secretion over the conventionally applied biomaterials, alginate and collagen. Co-culturing with human umbilical vein-derived endothelial cells decreased the central necrosis of islets. Culturing in both 3D gels (without printing) and the printed construct showed similar viability on days 1 and 5.	[74]
Cornea	Laser-assisted bioprinting (LaBP)	Human embryonic stem-cell-derived limbal epithelial stem cells (hESC-LESC), human adipose-tissue-derived stem cells (hASCs)	Recombinant human laminin and human sourced collagen I	The printed hESC-LESCs retained an epithelium-like structure and showed apical expression of CK3 and basal expression of the progenitor markers. After 7 days in vivo transplantation in the porcine organ, the 3D-bioprinted stromal structures showed interaction and attachment to the host tissue.	[75]

**Figure 4:**
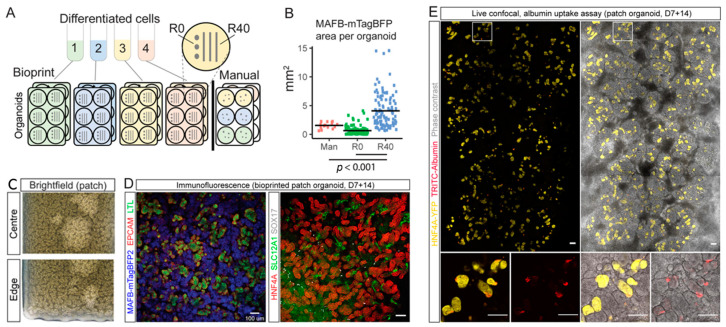
Cellular extrusion bioprinting improved nephron formation in kidney organoid, as compared to manual generation. (**A**) A schematic of the protocol for manual versus bioprinted kidney organoid formation (R40, R0). R40 and R0 were generated from 1.1 × 10^5^ differentiated iPSC (MAFB^mTAGBFP2^GATA3^mCherry^) cells, and manual organoids from 2.3 × 10^5^ cells. (**B**) Comparison of MAFB reporter area in manual and bioprinted kidney organoids. Larger area was observed in R40 organoids, suggesting greater nephron number formation. Bars indicate mean. R40-Man, *p* = 2.1 × 10^−5^, R40-R0, *p* = 2 × 10^−16^. (**C**) Uniform formation of nephron structures in the bioprinted kidney organoid patch, analyzed by brightfield imaging. (**D**) Nephrons showed expression of markers of proximal tubules (LTL (left panel; green) and HNF4A (right panel; red)), podocytes (mTagBFP2 (left panel; blue)), nephron epithelium (EPCAM (left panel; red)), distal tubule/loop of Henle TAL (SLC12A1 (right panel; green)), surrounded by interstitial endothelial cells (SOX17 (right panel; grey)). Analyzed by confocal immunofluorescence imaging. (**E**) Patch organoid was generated from proximal tubule-specific iPSC reporter line (where yellow fluorescent protein (YFP) was inserted under the control of the HNF4A promoter), following incubation in TRITC-albumin substrate. Live confocal imaging shows uptake of TRITC-albumin (red) into YFP-positive proximal tubules (yellow). Small panels below show higher magnification of the outlined areas, with and without phase-contrast overlays. Scale bars = 100 μm. Reproduced with permission from [68], copyright 2020 Springer Nature Ltd.

One of the major challenges in the transplantation of stem-cell derived kidney tissue has been the limited number of nephron structures. Takasato et al. reported the presence of approximately 100 nephrons within a transwell-cultured micro-mass kidney organoid that was initiated with 5 × 105 cells [76]. The human kidney, however, is estimated to contain approximately 1 × 106 nephrons [77]. Lawlor et al. compared extrusion-based 3D cellular bioprinting of kidney organoids with manual organoid generation [68]. In the manual approach, the organoids were generated by centrifuging cells to form an aggregate, and then the cells placed onto a transwell filter (Figure 4A). In the bioprinting approach, the organoids were generated by the automated deposition of cell pastes using a NovoGen MMX extrusion-based 3D cellular bioprinter. The researchers also varied the deposition ratio, that is, the ratio of the tip movement along the transwell surface to the volume of the deposited cell suspension, to obtain 2 different bioprinting conformations: (1) a single-point deposition (ratio 0, no tip movement at extrusion), and (2) a line of cells ~12 mm long (ratio 40, movement of 12 mm during extrusion) (Figure 4A). They used an MAFB^mTagBFP2^ reporter line for the cell paste and the MAFB-positive area from the fluorescence imaging of the resultant viable organoids as a surrogate for the nephron number [78,79]. They found that despite starting with a smaller cell number of 1.1 × 10^5^, the bioprinted R40 organoids contained a greater area of glomeruli, as compared to the manual organoids that had been initiated with 2.3 × 10^5^ cells (Figure 4B). In addition, they further examined the distribution and functionality of the patch organoid. With a deposition ratio = 30, they extruded the cell paste, containing approximately 4 × 10^5^ cells, across a total field of approximately 4.8 × 6 mm. They observed the uniform distribution of the epithelial structures (Figure 4C) and the correctly patterned nephrons (Figure 4D) in the resulting patch. They also showed evidence for the functionality of the nephrons, by the TRITC-albumin uptake into the YFP-positive proximal tubules (Figure 4E). Given the potential of adapting the bioprinting deposition ratio to enable additional nephron formation and larger fields of kidney tissue, further studies could investigate the bioprinting parameters for maximizing the nephron numbers to align with their human counterpart, the long-term formation of the nephron in the resulting organoid, and the functionality of the patches in vivo.

#### 3.1.3. Heart

**Figure 5:**
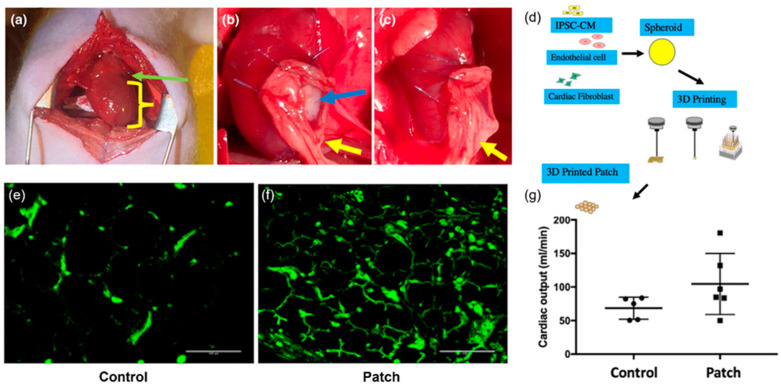
A scaffold-free approach to heart regeneration through printing cardiac spheroids containing human-induced pluripotent stem-cell-derived cardiomyocytes. (**a**) Surgical procedures of mouse myocardial infarction model. (**b**) Patch group: one cardiac patch (CP) and a natural omentum patch (OP) were sutured over the site of infarction. (**c**) One OP was sutured over the site of infarction. (**d**) Schematics of experimental setup. (**e**,**f**) vascularization of the infarcted area. Patch group vs. control group. Green: positive staining of the endothelial cell. Scale bar = 100 μm. (**g**) Cardiac output (mL/min). Patch group vs. control group. (CO: 104.6 ± 45.5 vs. 68.6 ± 16.4, *p* = 0.1). Reproduced with permission from [70], copyright 2019 John Wiley & Sons, Ltd.

There have been many efforts in the regeneration of heart tissue through cardiac stem-cell therapy. Many studies have printed cardiac tissue with a scaffold for implantation. The inclusion of a scaffold, however, has resulted in challenges related to mechanical properties, immunogenicity, and degradation. As previously mentioned, researches have been searching for the optimal scaffold. Moreover, Ong et al. proposed a novel method for bioprinting cardiac patches without a scaffold [69]. This scaffold-free approach involved printing cardiac spheroids containing human-induced pluripotent stem-cell-derived cardiomyocytes (hiPSC-CMs), fibroblasts (FB), and endothelial cells (EC). The cardiac tissue exhibited spontaneous beating and desired ventricular myocyte-like electrophysiological properties. Another in vivo implantation study examined the regenerative potential of this approach. Female Lewis nude rats were subjected to myocardial infarction (Figure 5a), and bioprinted cardiac patches were implanted and evaluated on the scar areas, in addition to their vascularization and cardiac functions (Figure 5b,c). The implantation group exhibited less scar area, higher vessel counts (Figure 5e,f), and a higher cardiac output than the control group (Figure 5g). However, since the heart rate of rats was different from the contraction rate of a human iPSC cardiac patch (CP) and the integration of the CP with a natural heart was not shown, further studies should investigate the long-term regeneration potential and the implant–host integration [70].

#### 3.1.4. Neurons and Central Nervous System

Neurons and glial cells are fragile and, thus, challenging for 3D printing. Previous studies have printed undifferentiated hiPSCs first and then allowed the cells to differentiate and self-assemble in brain organoids. It was reported that co-printing biomaterials and human neural stem cells (hNSCs) could encapsulate the stem cells, followed by their in situ expansion and differentiation into functional neurons and neuroglia [71]. As this method lacked control over the differentiation and the formation of brain constructs post-printing, there have been efforts to print neurons directly. Salaris et al. demonstrated another approach based on the extrusion printing of cortical neurons and glial cells, with Matrigel and alginate as the bio-ink. They showed the long-term survival of neurons, up to 70 days post-printing. Their functional analysis showed early and immature network activity [49]. Other researchers have also developed worked on the development of novel printing technology with fibrin-based bio-ink to print hiPSC-derived neural progenitor cells and observed early neuronal expression markers [72,73]. In addition, there have been advancements in the implantation of biocompatible scaffolds with neural stem cells (NSCs) and neural progenitor cells (NPCs). Hsieh et al. embedded NSCs in polyurethane (PU)-based thermos-responsive and biodegradable hydrogels, which repaired the nervous system function in zebrafish with brain injury, as demonstrated by a significant increase in hatching rate [80]. Recently, Joung et al. embedded spinal neuronal progenitor cells (sNPCs) and oligodendrocyte progenitor cells (OPCs) in a bioprinted scaffold, and as a result of their multicellular approach, they observed the axon propagation of printed sNPCs in the scaffold [54].

#### 3.1.5. Others Approaches for Pancreatic and Corneal Applications

Furthermore, bioprinting with stem cells has other applications, including in pancreatic and corneal diseases. Kim et al. used a pancreatic-tissue-derived extracellular matrix to recreate a natural tissue micro-environment and increase islet function in the 3D constructs [74]. They also observed a decrease in the necrosis of the islets when adding the human umbilical-vein-derived endothelial cells to the culture [74]. Sorkio et al. used human embryonic stem-cell-derived limbal epithelial stem cells (hESC-LESC) and human adipose-tissue-derived stem cells (hASCs) to print multilayer structures that resembled natural corneal tissue [75]. The implantation in a porcine corneal organ demonstrated an interaction with the host tissue and possible hASC migration [75].

### 3.2. Printing of In Vitro Models for Drug Development

The efficacy of personalized medicine is largely governed by the efficacy of the drug-development process, defined as the process through which potential drugs are identified, assessed, and optimized, prior to clinical trials [81]. Recent papers have explored the application of bioprinting technologies for drug development, especially in the development of 3D human cell organoids, organ-on-a-chip, and 3D-printed human cell assays. The development of such anatomically relevant 3D-bioprinted models for various tissues and disease states emphasizes the current status and future potential of bioprinting technologies in advancing drug development and resolving a key challenge in personalized medicine.

The primary contribution of bioprinting to drug development in personalized medicine has been through the development of more physiologically relevant models, which can expedite the drug development process, improve model reproducibility, and facilitate model customization. In comparison to conventional 2D models, 3D models have better capabilities for modeling cell–cell/matrix interactions and the spatial distributions of cells, thus improving the in vitro and in vivo correlations in clinical drug trials [82]. Based on this increased physiological accuracy, 3D-bioprinted models have been predicted to reduce resource demands and time intensity, as compared to the approximately USD 2.6 billion and 15 years required by current models and drug development systems to introduce a new drug to the market [83]. Furthermore, the bioprinting process provides significant control in the development of models (e.g., organoids) and, thus, is likely to improve model reproducibility and customizability in personalized medicine. This section highlights examples of 3D-bioprinted cardiac tissue and liver constructs and glioblastoma models.

#### 3.2.1. Cardiovascular Models for Drug Development

**Figure 6:**
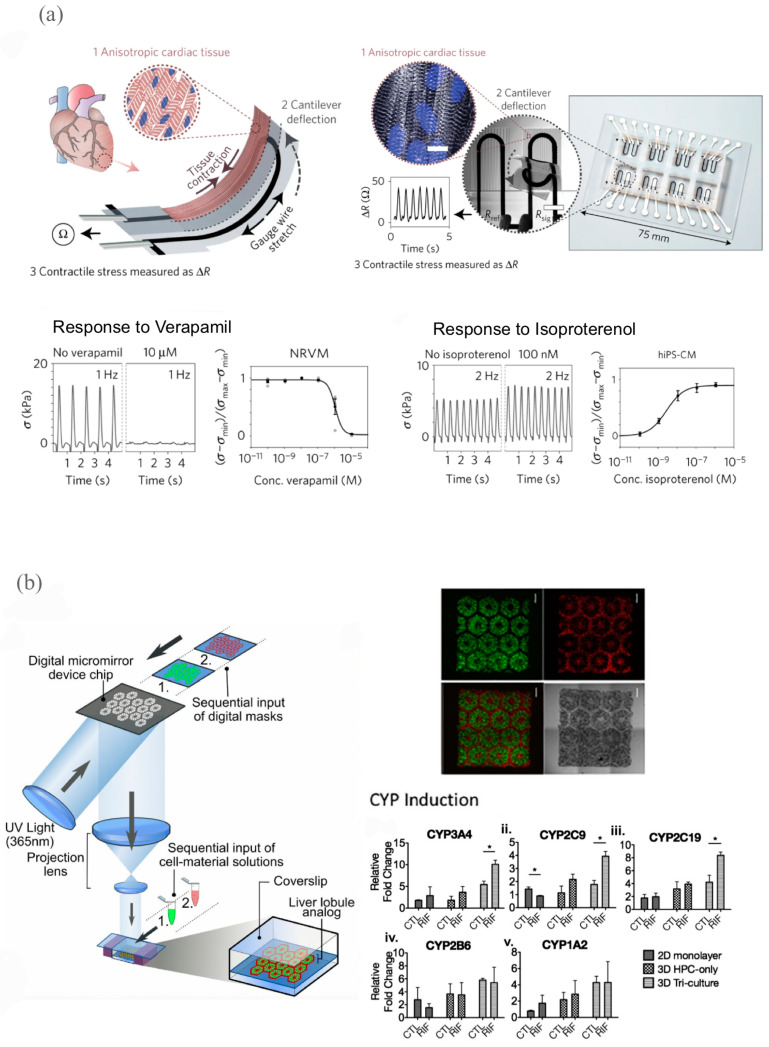
Bioprinted cardiovascular and liver models for drug development. (**a**) Schematic diagram of cardiac tissue model design with stress sensors (scale bars are 10 mm) and verapamil and isoproterenol dose-response plots below. Adapted with permission from [85]. 2016, Nature Publishing Group. (**b**) DLP-bioprinted hepatic model on left, fluorescence and bright field images of 3D-printed construct on top-right, and CYP induction plots on lower-right with asterisks indicating statistical significance with threshold of *p* < 0.05 (scale bars are 500 mm). Adapted with permission from [86]. 2015, S. Chen.

Cardiovascular diseases represent the leading cause of death in the United States, and cardiovascular drugs have clinical trial failure rates as high as 80%, which has established cardiovascular drug development and discovery as a key area for advancement [19,20]. This high failure/market-retraction rate has been attributed to the lack of physiologically natural 3D micro-environments in the cell cultures used to evaluate cardiotoxicity, as well as to the disproportionate effects of these drugs on various populations (e.g., ethnicities, the elderly, etc.) [82,84]. Therefore, representative and personalized biomimetic screening platforms for cardiovascular drugs are urgently needed for advances in 3D bioprinting. Lind et al. introduced multi-material inkjet 3D-bioprinting to advance existing microphysiological systems, such as organs-on-chips, by condensing the multi-step lithographic development of these systems into a single manufacturing step (Figure 6a). [85]. The authors of this study developed multiple functional bio-inks based on factors, such as piezoresistance, to construct micro-architectures that directed the self-assembly of laminar rat-derived cardiac tissues [85]. Researchers have similarly integrated non-invasive contractile stress sensors that enabled the electronic communication of contractile stress data and eliminated the laborious microscopy-focused designs involved in optical data communication, allowing data acquisition in cell-incubator environments [85]. Researchers observed that a 3D-bioprinted microphysiological device demonstrated inotropic responses to verapamil (L-type calcium channel blocker, which were similar to data from isolated whole postnatal rat hearts, demonstrating the model’s potential as a drug development platform [85]. The results of these preliminary studies and the continuous refinement of these engineered cardiac tissues point to bioprinting as a promising technology to advance personalized medicine.

#### 3.2.2. Liver Models for Drug Development

Liver models are especially relevant for drug development since drug responses can vary between individuals, and drug-induced liver injury is one of the most common causes for discontinuing clinical trials [82]. Furthermore, conventional human models are often costly and unreliable in terms of their translation to human studies due to differences in the hepatocellular functions of different species [87,88,89]. A recent study applied DLP technology to produce an in vitro 3D model of human-induced pluripotent stem-cell derived hepatic progenitor cells (hiPSC-HPCs) and nonparenchymal cells with micro-scale resolution (Figure 6b) [86]. The authors observed that the 3D-bioprinted triculture model demonstrated an increase in the anabolic and catabolic functions of the hiPSC-HPCs and key cytochrome P450 enzyme expression levels, as well as an improved drug induction/metabolism potential, as compared to conventional 2D-monolayer and 3D single-culture (HPC only) models [86]. This in vitro hepatic model and its facilitation of the hiPSC-derived hepatic cell maturation and functional maintenance in a biomimetic micro-environment signaled the significant potential for patient-specific drug development [86].

#### 3.2.3. Kidney Models for Drug Development

**Figure 7:**
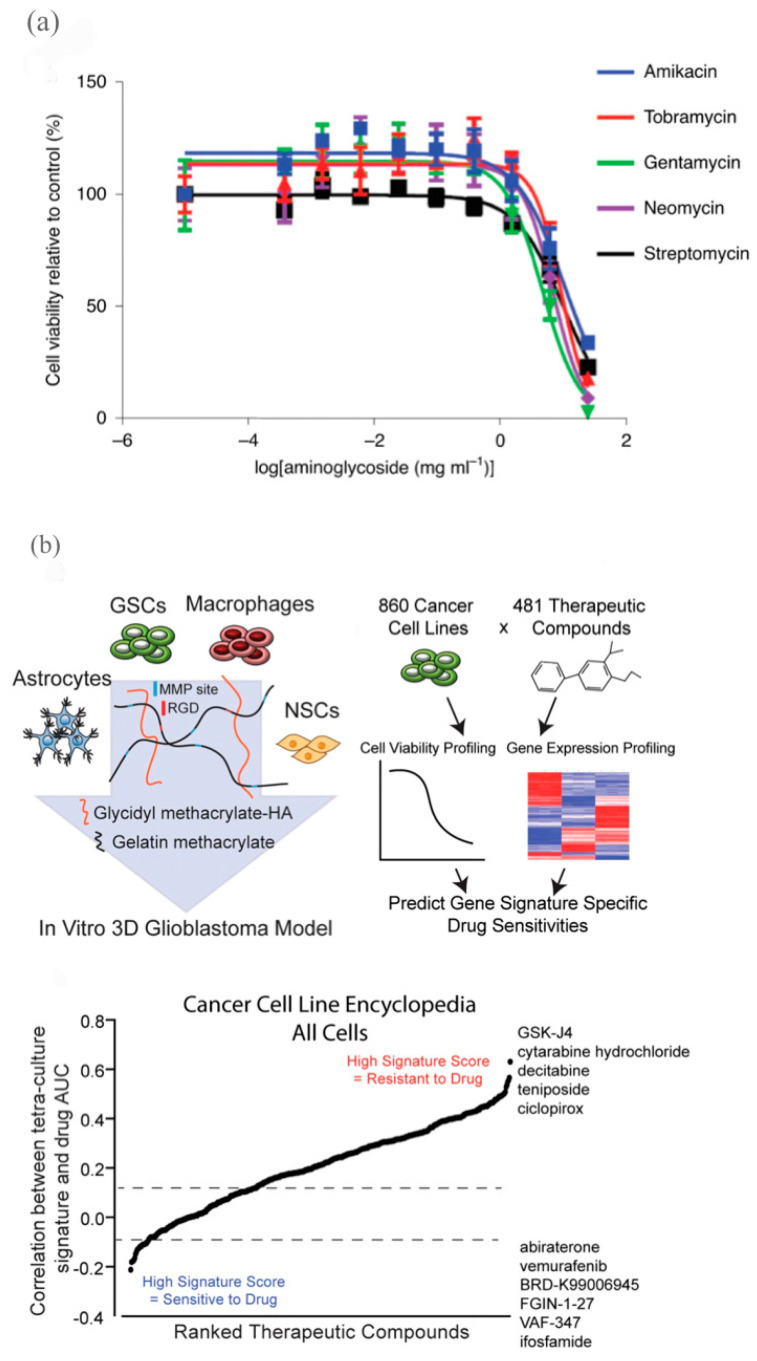
Bioprinted kidney and glioblastoma models for drug development. (**a**) Application of 96-well bioprinted organoids for testing viability in response to aminoglycoside antibiotics. The curves represent a non-linear fit for each compound, with *n* = 19 (amikacin), *n* = 24 (tobramycin), *n* = 30 (gentamycin), *n* = 30 (neomycin), *n* = 22 (streptomycin). Adapted from [68]. 2020, Nature Publishing Group. (**b**) Development of 3D glioblastoma model involving glioblastoma stem cells (GSCs), macrophages, astrocytes, and neural stem cells (NSCs) on left, methodology of evaluating drug sensitivity based on 3D tetra-culture gene expression signature from Cancer Cell Line Encyclopedia (CCLE) and the Cancer Therapeutic Response Platform (CTRP) datasets in middle, therapeutic efficacy prediction of drugs in CTRP dataset cancer cells using differentially expressed genes (as determined by RNA-sequencing) between 3D tetra-culture model and GSCs grown in sphere culture. Adapted from [92]. 2020, Nature Publishing Group.

Traditionally, 2D cell cultures used to assess nephrotoxicity in drug development have been unable to accommodate the 3D micro-environment of the adult human kidney, thus directing attention to the development of 3D models for the in vitro study of the organ, as well as for drug development and nephrotoxicity screening [90]. While bioprinted renal constructs have been challenged by the selection of appropriate cells and materials for bio-inks and by the development of complex renal structures, bio-inks such as a kidney-derived extracellular matrix and gelatin-fibrin hydrogels have demonstrated significant potential in recreating the in vivo kidney micro-environment [90]. Pluripotent stem-cell-derived renal progenitors have likewise been used to construct rudimentary multicellular structures, similar to those in vivo [91]. For example, Lawlor et al. utilized extrusion-based 3D bioprinting to deliver the rapid, high-throughput generation of kidney organoids with improved reproducibility and viability [68]. These organoids rivaled the morphology, the component cell-type, and the gene expression of organoids produced manually [68]. The automatic bioprinting of iPSCs into a 96-well-plate platform with a high number of viable and reproducibly patterned organoids was similarly assessed for high-throughput, drug-induced nephrotoxicity testing [68]. The authors treated the organoids with a series of nephrotoxic aminoglycosides and observed a concentration-dependent decrease in cell viability, which was consistent with observations of kidney injury in patients treated with aminoglycoside therapy (Figure 7a) [68]. The authors ultimately concluded that bioprinting was a feasible strategy for drug-testing in kidneys [68].

#### 3.2.4. Brain Models for Drug Development

Multiple groups have similarly applied 3D bioprinting to develop disease models to assay potential drugs. A recent study utilized DLP to develop an in vitro biomimetic tissue model for the glioblastoma tissue and micro-environment and to simulate immune interactions in the neural environment (Figure 7b). [92]. The authors identified that their bioprinted constructs with integrated macrophages were highly similar to patient-derived transcriptional profiles that were predictive of patient survival and the maintenance of stemness, invasion, and drug resistance [92]. Furthermore, the authors compared the gene expression data from the 3D tetra-culture model with the gene expression and drug sensitivity data from the Cancer Cell Line Encyclopedia and the Cancer Therapeutic Response Platform to derive predictions of drug sensitivity and resistance in the bioprinted construct based on its transcriptional signatures [92]. While these models were unable to completely recreate the disease state, more current models have been highly promising [92].

Preliminary studies indicated a significant potential for 3D bioprinting to advance personalized medicine. However, current constructs have been limited by the need for appropriate and accurate bio-ink materials and by the lack of consistency in differentiation and maturation protocols for induced pluripotent stem cells [24]. Furthermore, while a bioprinting resolution of nearly 200 µm has been achieved and demonstrated to be suitable for blood vessel models and organoids, higher printing resolution is required to produce fine capillary networks [32]. The density–viability–resolution trilemma has acknowledged the difficulty of producing a 3D-bioprinted structure that has a high cellular density (≥20 million cell/mL), a high cell viability (≥80%), and a high fabrication resolution (≤50 µm), simultaneously [93]. As 3D bioprinting advances in these sectors (as it undoubtedly will, given the field’s rapid progress and current attention), this novel technology could improve its capacity to model human tissues and advance personalized medicine.

## 4. Conclusions

Bioprinting enables the fabrication of tissue-like 3D constructs with tissue-level integrity by patterning living cells and biocompatible materials in a well-defined, layer-by-layer manner. There is no one-size-fits -all technique or material for bioprinting currently. The most impactful techniques have included inkjet printing, extrusion printing, laser-assisted printing, and stereolithography. The predominant materials have included natural (e.g., collagen) and synthetic (e.g., PEG) hydrogels.

Bioprinting stem cells is a promising method for personalized tissue regeneration. Most studies have been able to print hiPSCs or hiPSC-derived cells with high viability, differentiation, and proliferation, as well as subsequent tissue constructs with certain functionalities at the tissue level. Despite its promising future, bioprinting tissue has several key challenges to solve before it can be further evolved for regenerative implants. For example, it is still difficult to directly print small tissues (e.g., capillary vessel, 8–10 µm in diameter). Therefore, to fully reconstruct the cellular micro-environment, bioprinting techniques with higher resolution and better structural integrity need to be developed in order to maintain highly detailed, microscopic features during printing. In addition, while the short-term integration and vascularization were demonstrated in the preliminary in vivo studies on implantation, the long-term immunogenicity, integration, and maturation of implanted bioprinted tissues has yet to be determined. As allogeneic transplants often cause graft-versus-host disease (GvHD), we suspect there is a demand to comprehensively understand the host-immune response to the bioprinted grafts in future preclinical and clinical trials.

In addition to the in vivo applications, multiple groups have similarly developed 3D-bioprinted models to recreate natural tissue activity in vitro to expedite drug development. Such efforts have attracted significant attention from the pharmaceutical industry and regulatory agencies (i.e., FDA). Indeed, in early 2023, the FDA lifted the mandatory requirement of animal studies before human trials in the drug discovery pipeline. This reveals the immense potential for bioprinting-mediated in vitro drug development. With more research into reproducibility, quality control, and automation, we predict that bioprinting can be standardized for next-generation preclinical drug testing in the near future. It may also be possible to fluidically or physically connect bioprinted tissues to simulate body-level drug responses.

In conclusion, after two decades of development, 3D bioprinting has become a powerful yet versatile technique to generate tissue-like 3D constructs. With further technical development and additional biological validation, 3D bioprinting has the potential to transform the design of next-generation personalized medicine.

## Figures and Tables

**Table 2:** Summary of available natural and synthetic bio-inks.

Bio-ink Material	Description	Advantages	Disadvantages	References
Alginate	Natural negatively charged polysaccharides from brown algae	Non-immunogenic when implanted in vivo High biocompatibility Capable of transporting oxygen, nutrients, etc.	Lack of cell adhesion Poor printability Unpredictable biodegradability	[14,35,36,37]
Agarose	Polysaccharide obtained from seaweed	High cell viability	Poor support and limited cell growth	[35,38]
Collagen	Structural protein in the extracellular matrix	Easily obtainable from skin and connective tissues of organisms Relatively strong 3D structures	Poor mechanical properties, unless cross-linked Low mechanical strength Unpredictable viscosity and elastic modulus	[39,40]
Nanocellulose	Cellulose that can be derived from biomass, bacteria, and marine sources	Non-cytotoxicity High-aspect-ratio Strong mechanical properties	May not be an accurate model for human cells as we do not produce cellulase to be biodegraded	[41]
PEGDA	Synthetic polymer used for hydrogel fabrication and UV curing	Highly biocompatible Non-toxic and non-immunogenic Capable of photopolymerization High mechanical strength	Material can be brittle and rigid	[42]
Pluronic^®^	Synthetic polymer-poloxamer	Excellent printability Temperature-responsive gelation	Biocompatibility is not sufficient for long-term cell survival	[43,44]

Note: More information is provided in Table 2 in reference [13].

## Data Availability

Not applicable.
